# Gender representations and portrayal of adults in children’s television advertising: content analysis of prime cartoon channels in India

**DOI:** 10.3389/fpsyg.2023.1234678

**Published:** 2023-09-07

**Authors:** Kamalakannan Ravishankaran Chitra, Nakkeeran Senthilkumar

**Affiliations:** Department of Management Studies, College of Engineering, Anna University, Chennai, India

**Keywords:** children, adults, television advertising, gender stereotypes, content analysis, Asian countries, gender portrayals, India

## Abstract

**Introduction:**

Gender stereotyping in television advertising is a universal phenomenon and has the potential to influence children’s perceptions of gender roles. Despite India’s enormous child population, gender representation in television advertising is hardly researched.

**Methods:**

This study was conducted using a content analysis methodology to examine gender stereotypes in children’s television advertising in India. A total of 189 unique advertisements were selected from six prime cartoon channels, namely Nickelodeon, Disney Channel, Hungama, Cartoon Network, Discovery Kids, and Pogo TV. A stratified constructed sample of 319 central figures from 102 hours of TV viewing was collected over two consecutive weeks in November 2018 during children’s prime-time viewings.

**Results:**

This study examines gender role cues in children’s television advertising using McArthur and Resko’s coding method. The sample was subsequently analyzed using chi-square statistics, and the findings were contextualized and compared with those from other Asian nations. The results indicated that men significantly dominate (voice-over, product authority, autonomous roles, fact-based arguments, other products, end comments, pleasure, and practical rewards), whereas females are stereotyped (dependent roles, product users, portrayal of domestic products in domestic settings, opinion-based arguments, and self-enhancement rewards).

**Discussion:**

However, the results reveal a reduction in certain stereotypical aspects, such as a significant increase in women performing voice-overs and portraying characters with product authority and autonomy, while men exhibit increased involvement with domestic products and rewards such as self-enhancement, practical, and pleasure rewards. The theoretical (social learning and role congruity theory) and practical implications for advertisers and marketers are discussed based on these findings.

## 1. Introduction

Observational learning from symbolic models, such as those portrayed on television, can profoundly influence the perception of gender-appropriate behaviors in adults and children (Mayes and Valentine, 1979; Peirce, 1989; Smith, 1994). In that respect, advertising has a significant influence on our lives; it offers a wealth of information to children and, simultaneously, provides financial support for programming aimed at them (Smith, 1994). Vinacke (2010) defined stereotypes as a “set of concepts or beliefs on a social category,” whereas gender stereotypes are beliefs with certain attributes that differentiate between men and women (Ashmore and Del Boca, 1981). These stereotypes pose a problem because of their potential impact on how children view themselves and others (i.e., gender socialization) when competing messages and values concerning gender roles are misinterpreted or absent (Smith, 1994). According to social learning theory (Bandura, 1969), gender socialization can lead children to believe that certain roles are gender-specific through the observation and imitation of others. It suggests that children closely observe and imitate the attitudes, values, and behaviors of same-gendered models to determine what it means to be male or female (Bandura, 2001; Bandura and Bussey, 2004). The theory also predicts that television viewing influences children’s knowledge of gender (Bandura, 2001; Bandura and Bussey, 2004). Moreover, television is a highly impactful social construct that is crucial in shaping individuals’ perceptions of gender roles. These gender roles are defined differently across diverse societies and cultures. Role congruity theory asserts that individuals’ conformity with their expected roles depends on the stereotypes of their gender (Eagly and Karau, 2002). When these stereotypes are manifested in contradictory ways in real-world advertisements, it results in portrayals of odd behavior and a lower level of acceptability within contemporary social norms. Moreover, Browne (2013) states that children can learn archaic gender roles that are reinforced later in adulthood because of their early repeated exposure to such negative stereotypes in advertising. Therefore, negative stereotypes in advertising have attracted concern from parents, educators, and members of the advertising industry, as there is a possibility that children may acquire these stereotypes or be influenced by them. Additionally, the issue of stereotypes in advertising on children’s television is a social concern that warrants investigation.

In India, children under 14 years of age account for 30.76% of the total population [MoSPI (Ministry of Statistics and Programme Implementation Government of India), 2018]. India has the second largest television market after China, making it a desirable market for children’s content (Jaggi, 2017). This television market caters to 188 million households, 98% of which own a single TV [KPMG (Klynveld Peat Marwick Goerdeler), 2018]. Moreover, this television market is expected to grow from $10.11 billion in 2018 to $17.60 billion in 2023. In 2018, television advertising was the most significant contributor to the media industry, generating $3.47 billion (IBEF (India Brand Equity Foundation), 2018). According to KPMG (Klynveld Peat Marwick Goerdeler) (2018), among the various sectors, fast-moving consumer goods, auto, and telecom sectors contributed more than two-thirds of their expenditures solely to TV advertisements. According to the Broadcast Audience Research Council [BARC (Broadcast Audience Research Council), 2018a], 25% of the 211 million children aged 2–14 in India had a 20% share of viewership on children’s channels. Furthermore, Indian children have an annual purchasing power of Rs 22,594 crore, which is more than the combined annual gross domestic product of 50 smaller countries (Turner-NewGen Report, 2016). A study by Kapoor and Verma (2005) investigated Indian children’s cognitive responses to television advertising. Nearly 64.30% of children aged 3 to 6 were more interested in television advertisements than in television programming as they perceived the content to be realistic. The study also revealed that as children grow older, their cognitive understanding of television advertisements increases and their preferences shift toward television programming. Consequently, children over the age of eight recognize the advertiser’s persuasive intent and are well-informed about the product’s features, price, and availability. These mentioned characteristics indicate the importance of television in Indian culture and the significance of gender portrayals in local and international advertising. Gender representation is crucial because it helps us understand the importance of gender equality in today’s society. Therefore, it is imperative to study and improve gender portrayals in children’s television advertising.

### 1.1. Gender portrayals in television advertising

In 1975, McArthur and Resko developed a coding framework to analyze advertisements and their central characters for gender stereotyping in the United States. The authors rated central characters in advertisements based on gender, credibility basis, role, location, product argument, rewards, consequences of not using the product, and product type (McArthur and Resko, 1975). This study found that men and women were portrayed stereotypically in television advertisements. Researchers, including Gilly (1988), Goffman (1976), and Neto and Pinto (1998), have since utilized different coding approaches to examine gender roles in comparative studies. With the exception of Goffman, they all used McArthur and Resko’s framework as a foundation. The McArthur and Resko coding scheme, with its clearly defined key variables, paved the way for subsequent content analysis studies and has since become the primary research method for examining gender roles in advertising (An and Kim, 2007). It is perhaps rare to see a coding scheme endure for so long, despite the technique of content analysis being descriptive and deemed “expensive” research in data gathering and analysis (Furnham and Paltzer, 2010). Three major reviews of over 60 studies in this field have used the McArthur and Resko coding scheme (Furnham and Mak, 1999; Furnham and Paltzer, 2010; Furnham and Lay, 2019).

Previous studies on adult-focused television advertising have indicated that gender stereotypes are pervasive in many countries (Furnham and Mak, 1999; Furnham and Paltzer, 2010; Furnham and Lay, 2019). Some researchers argue that gender stereotypes are widely shared, whereas others believe they are country specific. Fewer studies have employed the McArthur and Resko coding scheme on samples of children’s advertisements than on samples of adult-focused advertisements in Western, Asian, and European countries. Most research conducted on children’s television advertising is cross-cultural, thus limiting single-country analysis. Chu and Mcintyre (1995) analyzed sex role stereotypes in children’s cartoons in Hong Kong and observed that male characters outnumbered female characters by a ratio of 2:1, whereas female characters were more prevalent in Japan than in the United States and the United Kingdom. The majority of the characters conformed to gender-based stereotypes. A cross-cultural study by Furnham et al. (1997) found that central figures and voice-overs were more often portrayed and performed by men than women in both American and British advertisements. A content analysis of children’s television advertisements from China and the United States (Ji and McNeal, 2001) revealed that Chinese children’s commercials reflect traditional cultural values, a transfer of authority from the older to younger generation due to the one-child policy, and Western values. Leaper et al. (2002) investigated gender-stereotyped content in children’s cartoons and found that male characters were overrepresented in traditional adventure and comedy genres, and that physical aggression was more frequently used by male characters than by female characters in the former genre. Similarly, Furnham and Saar (2005) compared British and Polish samples of advertisements and found that men predominantly portrayed central figures in advertisements geared toward children.

Wong and Chan (2006) evaluated portrayals of gender roles and gender traits in Chinese children’s television advertising and found that adult-focused advertisements were less gendered than those aimed at both children and adults. Males dominated voice-overs and were frequently portrayed in independent roles, while females played relationship roles. Moon and Chan (2002) analyzed gender portrayals in Hong Kong and Korea and found that the degree of gender stereotyping in Hong Kong advertising was similar to that in Korean advertising. Neto and Furnham (2005) compared television advertising targeted at children in Portugal, the United States, and the United Kingdom and found similarities and differences across the countries in gender role stereotypes. Sixsmith and Furnham (2010) investigated food advertising directed at adults and children on British television and found that child-focused advertising primarily featured male characters presenting health claims and scientific information, and in leisure settings. However, adult-focused advertisements primarily featured female celebrities presenting price/value information and in a retail store setting. Rubie-Davies et al. (2013) examined the depiction of men, women, and ethnic minority groups in 3000 television advertisements in New Zealand. The depictions of ethnic minorities were found to be stereotypical: Maori and Pacific Islanders were frequently portrayed in a negative manner, while some Asians were only depicted in advertisements for banking and finance.

By contrast, few research articles have examined the gender portrayals targeted at children in India. Bakir (2013) analyzed characters’ self-presentation behaviors on children’s networks in India and the United States using Goffman’s (1976) coding scheme. Relevant elements, such as voice-overs, indicated gender differences, and male voice-overs were used in more than half of the advertisements in both countries. Jaggi (2017) explored the role of cartoons and strongly indicated that Indian children consume a large amount of gender-stereotypical content. Furthermore, prior Indian studies on adult-focused advertisements indicated that the portrayal of men and women was gender stereotyped in television advertisements (Gupta and Jain, 1998; Munshi, 1998; Jha-Dang and Vohra, 2005). A detailed study by Jha-Dang and Vohra (2005) found that women appeared more frequently in advertisements for household and personal or beauty products, whereas men appeared more frequently in advertisements for automotive products, financial services, and electronic products. On adult-focused television advertising, Das (2011) and Moorthi et al. (2014) concluded that while gender stereotyping persisted on Indian television, a positive and modern role shift had been observed in women’s portrayals.

Choudhary and Roy (2022) analyzed 212 television advertisements from 1990 to 2020 to identify differences in product type and audience. The authors observed that from 2010 to 2020, advertisers used emotive and informative appeals to entice both adults and children by using children as the central theme of their advertisements. Similarly, Chitra and Senthilkumar (2022) analyzed age-related stereotypes in Indian children’s television advertising for 189 visual central characters using the McArthur and Resko coding technique. In television advertisements, both younger and middle-aged women were portrayed in a visually stereotypical manner, while middle-aged men were portrayed as authoritative characters, highlighting that age-related stereotypes persist. A few progressive attributes were observed, where advertisers attempted to use middle-aged women for alleged appeal, younger women in autonomous roles, and middle-aged men for self-enhancement products.

### 1.2. Research gap and study objective

This study highlights the following research gap: While earlier studies have addressed gender roles in adult-focused television advertising in India, research on children’s television advertising is conspicuously absent, as is the use of the preferred McArthur and Resko coding system for analyzing gender stereotyping. Moreover, the last gender data on adult-focused television advertising were obtained in 2004 (Das, 2011), where six variables from this coding system were investigated. Consequently, little is known about the changes or developments in societal gender roles presented in children’s television advertising relative to adult-focused television advertising based on the 2004 data (Das, 2011). This study evaluates the extent of stereotyping using data from children’s television advertising obtained in 2018. In addition, exploring additional variables within a single country (e.g., product argument, end comments, and reward) may predict significant differences in gender role portrayals. Thus, inclusion of these additional variables within the coding scheme should contribute to a more comprehensive picture of gender research in South Asian countries, particularly India, which has traditionally been dominated by studies conducted in Western and developed nations.

The primary objective of this study is to examine whether gender role cues are present in children’s television advertising by addressing the frequency of men and women appearing in television advertisements using the McArthur and Resko coding scheme. In addition, this study contextualizes the findings through comparisons with other Asian countries, thereby extending the breadth and depth of the research. This study employs established research methodologies and procedures and adheres to them as precisely as possible to permit comparisons of gender role portrayals in Indian television advertisements with those in other Asian nations. Consequently, children were not coded as central figures to prevent conflict when comparing primary source data with other Asian studies that overwhelmingly employ adults as central figures.

### 1.3. Research hypotheses

Although the exact nature of gender stereotyping varies slightly across cultures, evidence has shown that it is much more prevalent in traditional Asian countries (Neto and Pinto, 1998; Furnham and Mak, 1999; Furnham and Paltzer, 2010). Men outnumber women in television advertisements, according to the majority of Asian studies (Furnham and Mak, 1999; Furnham and Paltzer, 2010). Several Asian studies have found that men were featured more frequently in voice-overs, while women were portrayed visually in television advertisements (Bresnahan et al., 2001; Furnham and Imadzu, 2002; Kim and Lowry, 2005; Das, 2011; Prieler and Centeno, 2013; Prieler et al., 2015). Regarding the nature of the portrayals, females were more frequently depicted at home and as consumers of domestic products. By contrast, men were more likely to be seen in the workplace or outdoors and were associated with automotive accessories, sports, alcoholic beverages, and technology products. In many Asian countries, men tend to be depicted in autonomous roles, whereas women are typically portrayed in dependent roles but less frequently in working roles (Furnham and Mak, 1999; Uray and Burnaz, 2003; Furnham and Paltzer, 2010; Lim and Furnham, 2016). Similarly, advertisements frequently portray men as authoritative figures and women as consumers of the advertised products. According to Furnham and Mak (1999) and Furnham and Paltzer (2010), men are more likely to portray pleasure rewards, end comments, and fact-based arguments, whereas women are depicted as preferring self-enhancement rewards, few or no end comments, and opinion-based arguments when purchasing or utilizing a product or service.

Previous research in Western and Asian nations has uncovered consistent underrepresentation and stereotypical portrayals of women in television advertisements. Although India has a large child population, there is a dearth of research on the gender representation of children in television advertisements. This study seeks to close the gap in knowledge on gender representation and enhance our understanding of children’s television advertising in India. The research questions and 10 hypotheses are based on previous research findings from different Asian nations.

The following are the research questions: How are Indian men and women portrayed in children’s television advertisements based on gender differences? Has there been a change in how men and women are represented in children’s television advertising in India compared with other Asian countries?

Based on the previous research on gender roles in advertising, this study proposes the following hypotheses:

*H*1: Males show predominance over females in Indian television advertisements.

*H*2: Males are more likely to be represented through voice-overs, whereas females are more likely to be represented visually.

*H*3: Males are shown more often as product authorities, whereas females are shown more often as product users.

*H*4: Males are more likely to perform autonomous roles, while females are more likely to perform dependent roles.

*H*5: Males are represented in narrator roles more often than females are because of perceived autonomy.

*H*6: Males are shown more often in outdoor/occupational settings, whereas females are shown more often in domestic/indoor settings.

*H*7: Males are more likely to make fact-based arguments, whereas females are more likely to make opinion-based arguments.

*H*8: Males are more likely to be associated with other products, whereas females are more likely to be associated with domestic products.

*H*9: Males are portrayed more frequently as offering pleasure rewards, whereas females are portrayed more frequently receiving self-enhancement rewards.

*H*10: Males are more likely to make end-comments than females.

Most Asian research on adult-focused television advertising supports H2 (mode of presentation), H3 (credibility basis) and H4 (role), which propose that male characters often perform voice-overs and are portrayed as product authorities and in autonomous roles, while females are often presented in a visual context and are portrayed as product users and in dependent roles (For H2: Malaysia, Taiwan, and Japan; Bresnahan et al., 2001; India; Das, 2011; Japan; Furnham and Imadzu, 2002; Korea; Kim and Lowry, 2005; the Philippines; Prieler and Centeno, 2013; Hong Kong, Japan, and South Korea; Prieler et al., 2015; For H3: Furnham and Mak, 1999; Furnham and Imadzu, 2002; Furnham and Paltzer, 2010; Malaysia; Lim and Furnham, 2016; Turkey; Uray and Burnaz, 2003; For H4: Japan; Arima, 2003; Lim and Furnham, 2016; Saudi Arabia; Uray and Burnaz, 2003; Nassif and Gunter, 2008). Similarly, it is expected that women, compared with men, are portrayed in domestic settings (H6) and are more closely associated with domestic products (H8); several Asian studies have found this to be true in adult-focused television advertising (Bresnahan et al., 2001; Furnham and Imadzu, 2002; Uray and Burnaz, 2003; Kim and Lowry, 2005; Das, 2011; Prieler et al., 2015).

H1 (sex), H7 (product argument), H9 (reward type), and H10 (end comment), have been tested in several Asian countries in the context of adult-focused television advertising (Furnham and Mak, 1999; Furnham and Paltzer, 2010); however, the four hypotheses based on the McArthur and Resko coding method (H5: narrator role, H7: product argument, H9: reward type, and H10: end comments) have not been evaluated for gender representation in Indian television advertising for either adult-focused or children’s channels. For instance, in several Asian countries, H5 (narrator role) has been hypothesized and tested, indicating that males have more autonomy in narrator roles than do females (Furnham and Imadzu, 2002; Uray and Burnaz, 2003; Kim and Lowry, 2005). Additionally, the remaining hypotheses (H1, H2, H3, H4, H6, and H8) have been examined in a previous Indian study using 2004 data on adult-focused television advertising (Das, 2011), although not exhaustively in children’s television advertising.

## 2. Methodology

### 2.1. Cartoon channels and advertisements

According to the Turner-NewGen Report (2016), television is ranked as the highest form of media consumption at 97%, with cartoons being Indian children’s favorite genre. In 2018, the top six cartoon channels owned by international divisions, namely Nickelodeon, Disney Channel, Hungama, Cartoon Network, Discovery Kids, and Pogo TV, were selected from a sampling frame of 23 channels. These channels were chosen based on audience-share figures published in October 2018 in BARC’s weekly impressions. According to BARC (Broadcast Audience Research Council), (2018b), Nickelodeon, Pogo TV, Discovery Kids, Cartoon Network, Disney Channel, and Hungama received 58, 56, 54, 50, 45, and 43% of viewership, respectively. Moreover, a substantial proportion of the viewers of these channels are children aged 2–14 years. The sample taken from these six cartoon channels represents the media consumption of the Indian population for the following reasons. First, these channels account for most of the media consumption of child audiences, ensuring sample representativeness. Second, compared to previous studies (Furnham et al., 1997; Furnham and Saar, 2005; Neto and Furnham, 2005; Sixsmith and Furnham, 2010), the sample of advertisements and chosen timeframe represent a sufficiently large sample of children’s television. Third, all six channels can be viewed in four different languages (i.e., English, Telugu, Tamil, and Hindi). Given the language diversity in India, the available languages cover most of the regional child audiences in different states.

The data were collected consecutively for 2 weeks in mid-November 2018, and advertisements were recorded daily from 12:00 p.m. to 7:00 p.m. on weekdays (Monday to Friday) for 10 days, and from 12:00 p.m. to 8:00 p.m. on weekends (Saturday and Sunday) for 4 days. The selection of timeframes corresponded to peak viewership times. This was based on BARC (Broadcast Audience Research Council), (2018b) overall TV viewing by children (2–14 years of age) from week 1 to week 32 of 2018. This descriptive study employed a stratified constructed week sampling method. Similar to previous content analysis studies (McArthur and Resko, 1975; Furnham and Farragher, 2000; Arima, 2003; Uray and Burnaz, 2003; Nassif and Gunter, 2008), all repeated content and multiple copies of the same advertisement were discarded to ensure a full scope of unique advertisements; this was a common practice to ensure the feasibility of making comparisons with other studies. After disregarding the repeated content, the process yielded a sufficient total of 224 unduplicated advertisements. Similarly, advertisements featuring children, animals, fantasy characters that were not distinctly human, local/political advertisements, and central figures without an identifiable sex were excluded. Advertisements in English and Hindi for products marketed by national and international advertisers were included. This process yielded 189 separate advertisements from 102 h of TV viewing for further coding and analysis.

### 2.2. Variables and unit of analysis

The coding procedure used categories initially modeled on the well-established coding concepts by McArthur and Resko (1975), Manstead and McCulloch (1981), and Furnham and Voli (1989). Eight variables were selected for this study. Table 1 presents the modifications considered throughout the years in the categories of each variable. Many content analysis studies have followed the original coding categories of McArthur and Resko (1975), with certain modifications such as adding, splitting, and occasionally dropping categories (Furnham and Paltzer, 2010; Furnham and Lay, 2019). Accordingly, three reliability measures (average percentage agreement, intercoder reliability, and Krippendorf’s alpha reliability) were examined for all variables to ensure that the categories were well adapted to the Indian context. Unlike McArthur and Resko’s (1975) study, which coded only two central figures, this study adopted McArthur and Eisen’s (1976) coding of a maximum of four central figures, namely two central adults and two central voices, for each advertisement.

**Table 1:** Variables included in the study.

Variable	Description (Basis of Categories^c^)
Sex (99.79^a^; 1.0^b^)	Male, Female (McArthur and Resko, 1975; McArthur and Eisen, 1976)
Mode of presentation (99.79; 1.0)	Disembodied voice-over (bodiless), Wholly Visual (Manstead and McCulloch, 1981; Furnham and Bitar, 1993; Kay and Furnham, 2013)
Credibility basis (99.37; 0.97)	Product user (central figure depicted as a user of the advertised product), Product authority (central figure depicted as an expert concerning the product), Other (character depicted is neither user nor authority; McArthur and Resko, 1975; Manstead and McCulloch, 1981)
Role (99.37; 1.0)	Dependent (as a parent/caregiver, spouse, child, homemaker, gender/sexual object), Autonomous (as a professional or a researcher on the product like a leader or celebrity), Narrator (when the central figure describes the product being advertised; McArthur and Resko, 1975; Kay and Furnham, 2013)
Location (99.58; 1.0)	Domestic setting, occupational/outdoor environment setting, Other settings (leisure setting, store setting, unknown; McArthur and Resko, 1975; Furnham and Bitar, 1993)
Product argument (98.54; 0.97)	Opinion-based argument (personal comment about the quality of a product or service based on preference and experience), fact-based argument (tries to marshal facts explaining why the product or service is innovative or superior in some way), no argument (if the product alone is displayed; McArthur and Resko, 1975; Harris and Stobart, 1986)
Product type (100; 1.0)	Domestic products (food, home appliances and related products, and health and body-related products), toys/games, other products (products related to services and mobile applications, finance, entertainment, leisure, and sports; McArthur and Resko, 1975; Kay and Furnham, 2013)
Reward type (98.12; 0.96)	Social enhancement reward (opposite-sex approval, family or friends’ approval, social or career advancement), self enhancement reward (psychological improvement, attractiveness, cleanliness, or health), practical reward (saving time, labor, or money, useful, or functional), pleasure reward (when the use of the product yields satisfying or gratifying results), other rewards, no reward (McArthur and Resko, 1975; Harris and Stobart, 1986)
End comment (98.96; 1.0)	Present (a product slogan or the product name repeated at the end of the advertisement; Harris and Stobart, 1986)

a Percentages in parentheses indicate intercoder reliability scores for 319 units.

b Scores in parentheses indicate Krippendorf’s alpha reliability value for 47 units.

c The authors are listed in this order as “Original Author: Subsequent modifications in categories by other author(s).”

The unit of analysis was the central figure of the advertisement, resulting in a sample of 319 central figures from 189 separate advertisements. The central figure was defined as “any adult character or adult humanoid cartoon figure with a speaking role or the most prominent speaking lines or a visual appearance of at least 3 s in length in an advertisement” (Gilly, 1988; Milner and Higgs, 2012). When more than two central figures and prominent voices were present, those that appeared to be the most central or dominant were selected. Central figures with or without background visual characters were selected for voice-only advertisements.

### 2.3. Coding

For the content analysis, the researcher trained three coders (one man and two women) with fluency in two languages (English and Hindi). The coders received a coding sheet with a written description of all variables and attended a 3-h training session, during which the variables were clarified. Subsequently, 15 non-sample advertisements were used as a pretest for training purposes. Each coder coded these advertisements, and a researcher helped to resolve any discrepancies. Following the initial training, the researcher ensured that the three coders were blinded to the study’s hypotheses before they began coding the 189 recorded advertisements. Each coder independently agreed upon a maximum of four central figures per advertisement (two each for gender and voice) and then coded the nine variables. Following the initial coding, the three coders reconciled differences by reviewing the particular advertisement until an agreement was reached. When the coders disagreed, they contacted the researcher for clarification, and the agreed coding was pooled for data analysis. Among the 62 coding discrepancies, 18 and 14 related to reward type and product argument, respectively.

### 2.4. Coding reliability

The three coders individually coded 2,871 items for 319 central figures and 9 variables, for a total of 8,613 coding decisions. The average percentage of agreement between the three coders was 99.28%. This was calculated for the 319 units using the following formula: number of agreements/number of agreements + number of disagreements × 100. As presented in Table 1, the inter-coder reliability score for the 319 units was more than satisfactory for all variables. Since more than two coders were used, Krippendorf’s alpha reliability value was calculated for a pilot sample of 47 units (i.e., 14.73% of the total sample), which were chosen using a random sampling method (Riff et al., 2013). The reliability values presented in Table 1 revealed that the alpha coefficient values were reasonably high (greater than α = 0.80) for all variables at a 95% confidence interval (Hayes and Krippendorff, 2007).

### 2.5. Data analysis

Data were analyzed using IBM SPSS software. The majority of content analysis studies accomplish their objectives using means, proportions, and simple frequency counts, whereas 37% of these studies analyze content data predominantly using the chi-square statistical technique (Riff et al., 2013). Therefore, all hypotheses were tested using the chi-square test. If the value of p was less than 0.05, the chi-square was significant; in this study, the difference was highly significant at 0.01 and 0.001 levels for most variables. After excluding 47 units considered in the reliability test, the final sample yielded 272 central figures.

## 3. Results

The sex of the central figures concerns the portrayal of adults who exhibit a dominant presence, either visually or vocally, or both. Although males marginally outnumbered females (51.10% vs. 48.90%) in the sample of 272 central figures, no significant difference occurred in the sex variable (χ^2^ = 0.132, d.f. = 1; n.s). Thus, H1 is not supported (Table 2). The term “mode of presentation” pertains to how a central figure is perceived, either through whole visual representation or through voice-over. Approximately 56.8% of males were presented through voice-overs, whereas 77.4% of females were depicted visually. Single chi-square analysis for male voice-overs was relatively high (χ^2^ = 22.028, d.f. = 1, *p* < 0.001) compared to that of female visual portrayals (χ^2^ = 11.344, d.f. = 1, *p* < 0.001). Thus, H2 is supported (Table 2).

**Table 2:** Percentage of adult males and females by coding category.

Content categories	Male	Female	Total N (%)	Chi-Square^b^ χ^2^
	N (%)	N (%)		
Sex^a^ (H1)	139 (51.10)	133 (48.90)	272 (100)	0.132, n.s.
Mode of presentation (H2)				
Disembodied voice-over	79 (56.80)	30 (22.60)	109 (40.07)	22.028^***^
Wholly visual	60 (43.20)	103 (77.40)	163 (59.93)	11.344^***^
Credibility basis (H3)				
Product user	23 (16.50)	85 (63.90)	108 (39.71)	35.593^***^
Product authority	104 (74.80)	47 (35.30)	151 (55.51)	21.517^***^
Others / none	12 (8.60)	1 (0.80)	13 (4.78)	9.308^**^
Role (H4; H5)				
Dependent	16 (11.50)	64 (48.10)	80 (29.41)	28.800^***^
Autonomous	123 (88.50)	69 (51.90)	192 (70.59)	15.188^***^
Narrator	77 (68.00)	37 (32.00)	154 (56.62)	14.035^***^
Location (H6)				
Domestic / indoor setting	32 (23.00)	68 (51.10)	100 (36.76)	12.960^***^
Occupational / outdoor	17 (12.20)	22 (16.50)	39 (14.34)	0.641, n.s.
Others	90 (64.70)	43 (32.30)	133 (48.90)	16.609^***^
Product argument (H7)				
Opinion based	16 (11.50)	74 (55.60)	90 (33.09)	37.378^***^
Fact-based	100 (71.90)	45 (33.80)	145 (53.31)	20.862^***^
No argument	23 (16.50)	14 (10.50)	37 (13.60)	2.189, n.s.
Product type (H8)				
Domestic products	97 (69.80)	116 (87.20)	213 (78.31)	1.695, n.s.
Other products	32 (23.00)	13 (9.80)	45 (16.54)	8.022^**^
Toys and games	10 (7.20)	4 (3.00)	14 (5.15)	2.571, n.s.
Reward type (H9)				
Social enhancement rewards	6 (4.30)	2 (1.50)	8 (2.94)	2.000, n.s.
Self enhancement rewards	52 (37.40)	91 (68.40)	143 (52.57)	10.636^**^
Practical rewards	32 (23.00)	17 (12.80)	49 (18.01)	4.592^*^
Pleasure rewards	40 (28.80)	19 (14.30)	59 (21.69)	7.475^**^
Other rewards	2 (1.40)	3 (2.30)	5 (1.84)	0.200, n.s.
No rewards	7 (5.00)	1 (0.80)	8 (2.94)	4.500^*^
End comment^c^ (H10)				
Present	105 (75.50)	59 (44.40)	164 (60.29)	12.902^***^

^*^p ≤ 0.05, ^**^p ≤ 0.01, ^***^p ≤ 0.001.

a The relevant hypothesis for gender-based differences is given in parentheses.

b For all chi-square tests, the degree of freedom = 1.

c The sum of the percentages does not equal 100 because 108 central characters.

(39.71%) had no end comments.

The concept of credibility basis refers to the perceived credibility of the central figure portrayed in an advertisement. Approximately 63.9% of females were frequently portrayed as product users (χ^2^ = 35.593, d.f. = 1, *p* < 0.001), and 74.8% of males were frequently portrayed as product authorities (χ^2^ = 21.517, d.f. = 1, *p* < 0.001). Thus, H3 is supported (Table 2). The role of the central figure refers to the apparent position (either dependent or autonomous) of the dominant figure in everyday life, as depicted in the advertisements. The single chi-square values were highly significant, with 48.1% of females represented in dependent roles (χ^2^ = 28.800, d.f. = 1, *p* < 0.001) and 88.5% of males represented in autonomous roles (χ^2^ = 15.188, d.f. = 1, *p* < 0.001). The gender differences were found to be more robust, with 68% of males and 32% of females represented in the narrator role (χ^2^ = 14.035, d.f. = 1, *p* < 0.001). Thus, H4 and H5 are supported (Table 2).

Location refers to the place where the central figure is depicted in advertisements and is sometimes inferred from the people present. The location of the central figures in voice-overs was coded as “unknown” because their locations could not be determined. The gender differences were highly significant, with 51.1% more females than males being observed in domestic settings (χ^2^ = 12.960, d.f. = 1, *p* < 0.001). Although females outnumbered males by a slight margin (16.5% vs. 12.2%) in outdoor/occupational settings, it was numerically but not statistically significant. Thus, H6 is partially supported, as women are overrepresented in both domestic and occupational/outdoor settings (Table 2). Product argument refers to the central figure’s justification for utilizing the advertised product. The differences were highly significant, with 71.9% of males providing fact-based arguments (χ^2^ = 20.862, d.f. = 1, *p* < 0.001) and 55.6% of females providing opinion-based arguments (χ^2^ = 37.378, d.f. = 1, *p* < 0.001). Thus, H7 is supported (Table 2).

Product type refers to the type of product represented by the central figure in advertisements, such as domestic products, toys and games, or other products. Significance was generally observed, with 23% of males represented in the other product category (χ^2^ = 8.022, d.f. = 1, *p* < 0.005). Thus, H8 is partially supported because, although females marginally outnumber males (87.2% vs. 69.8%) in the domestic product category, it is numerically but not statistically significant (Table 2). For product users, rewards were those received by central figures (consumers), whereas for product authorities, rewards were those offered by central figures to consumers. Approximately 68.40% of the females were significantly more likely than males to receive self-enhancement rewards for using the product (χ^2^ = 10.636, d.f. = 1, *p* < 0.001). By comparison, males were significantly more likely than females to offer practical (23%) and pleasure rewards (28.80%) to the viewers for the advertised product (χ^2^ = 4.592 and 7.475, d.f. = 1, *p* < 0.032 and 0.006). Thus, H9 is supported (Table 2). End Comment indicates whether the central figure made a concluding remark or comment, either visually or vocally, at the end of the advertisement. The difference was highly significant, with twice as many males (75.5%) making an end comment as females (χ^2^ = 12.902, d.f. = 1, *p* < 0.001). Thus, H10 is supported (Table 2).

In summary, most of the hypotheses are fully supported (H2, H3, H4, H5, H7, H9, and H10). H6 and H8 are partially supported. However, H1 is not supported, as the findings indicate no significant difference in the frequency of male or female central figures.

## 4. Discussion

This study established that the gender-based hypotheses confirmed more differences than similarities (9 of 10 were significant), with 90% of the content categories portraying stereotypes of men and women in children’s television advertising. Compared with women, men twice as frequently portrayed autonomous roles, performed voice-overs, depicted product authorities, presented fact-based arguments and end comments, were associated with other products, and offered rewards that were mostly practical and pleasurable to consumers. Women were portrayed (1) twice as frequently as visual presenters, in domestic/indoor settings, and receiving rewards of self-enhancement for using the product; (2) three times as often as product users; and (3) four times as often in dependent roles and presenting arguments in the form of opinions.

In this study, men outnumbered women numerically but not statistically in gender representation (51.10% vs. 48.90%); these values mirrored the actual percentages of men and women (51.80% vs. 48.20%) based on Indian population data in 2018 [MoSPI (Ministry of Statistics and Programme Implementation Government of India), 2018]. The proportion of women in this study increased from 43.20% in 2011 (India; Das, 2011) to 48.90% in 2018. The percentage of female voice-overs increased from 10% (Das, 2011) to 22.6%, while the percentage of male voice-overs decreased from 80.40% (Das, 2011) to 56.80% in 2018. However, in this study, male voices were frequently used for advertising targeted at boys (primarily games), while female voices were utilized for cosmetics, personal care, and baby products. Regarding the credibility basis, this study found that the frequency of portraying a product authority increased for both genders (74.80% of males and 35.30% of females), representing values higher than those in a previous Indian study (11.50% of males and 7.8% of females; Das, 2011). Regarding product users, the proportion of women decreased from 70.3% (India; Das, 2011) to 63.90% in 2018. Moreover, the frequency of Indian women’s portrayal of autonomous roles was found to be increasing (51.90%), with a value higher than that in advertisements in children’s television studies by Moon and Chan (2002) in Hong Kong and Korea. Similarly, the frequency of women’s portrayals of dependent roles decreased from 52.4% (Das, 2011) to 48.10% in 2018.

The proportion of women portrayed in domestic/home settings was relatively high (51.10%), exceeding the previous Indian study by 13.8% (Das, 2011). Conversely, women outnumbered men in occupational/outdoor settings; however, this finding should be interpreted with caution, as categorizations varied from previous Asian studies and the location of all voice-overs was unaccounted for (i.e., placed in the unknown location category). Regarding product type, males were significantly associated with services, online apps, and financial products. By contrast, females were numerically but not statistically associated with female-oriented products (i.e., baby, body, household, and cleaning products). Additionally, the proportion of women appearing in domestic products increased from 52.8% (Das, 2011) to 87.20% in 2018. This study analyzed unexplored variables (i.e., argument, rewards, and end-comments) in Indian television advertising for either adult-focused or children’s channels, and found that men favored fact-based arguments (71.90%), while women favored opinion-based arguments (55.60%). Another noteworthy observation in this study was Indian males’ increased use of self-enhancement (37.40%), practical rewards (23%), and pleasure rewards (28.80%) in children’s TV advertisements. Similarly, it was found that 60.30% of the Indian advertisements portrayed on cartoon channels include an end-comment, with men significantly more likely (75.5%) than females to make an end comment for the advertised product.

The results of this study in India revealed that advertisers still embrace the traditional image of women, and portray them as being engaged in domestic or indoor activities but not preferred for endorsing products and services of high involvement (i.e., online apps and financial products). This case has been reflected in many variables, where women were predominately portrayed in advertising for products confined primarily to households, preferred giving opinions, endorsed self-enhancement products, and were projected in dependent roles as caregivers and nurturers. However, advertisers portray Indian men in authoritative and independent roles, such as voice-overs and experts in providing consumers with fact-based arguments, concluding comments, and practical and pleasurable rewards. This pattern only emphasizes that women are presumed to lack competence and prefer giving opinions, while men are still considered to hold greater authority in making final decisions in television ads. According to social learning theory, such a perception might be further conveyed to the audience, particularly children, by reinforcing the association of a specific gender with authority within a society (Bandura, 2001).

However, when comparing the results to those of Das (2011), the situation for Indian women in children’s television advertising appears to have marginally improved. Their presence has increased in variables dominated by men, such as voice-overs, product authorities, and autonomous roles, while it has decreased in variables dominated by women, such as product users and dependent roles. For instance, advertisers have attempted to portray women in professional settings, but their employment status is either downplayed or not indicated properly. Advertisers should begin to account for India’s shifting demographics and employ women in gender roles that reflect these evolving societal and cultural norms. Similarly, more preference has been given to female celebrities in children’s advertisements, primarily in sports and film, but with decorative portrayals for advertising domestic products. In contemporary India, advertisers must take into account the changing roles of men and women in the home and the workplace, and should aim to present messages that exhibit equitable gender preferences when targeting children.

### 4.1. Comparison with gender portrayals in other Asian countries

Although McArthur and Resko’s framework has indeed served as the foundation for more than 60 studies, the differences in content measures across cultures have been thoroughly researched, thus making comparisons with other Asian countries viable to a certain extent. Only advertisements from other Asian countries, such as Japan, Korea, Taiwan, Turkey, Singapore, Malaysia, Hong Kong, and the Philippines, can be used as a point of comparison because insufficient research exists on gender roles in advertising in South Asian nations, such as Pakistan, Bangladesh, Sri Lanka, and Nepal. In addition to Moon and Chan’s (2002) cross-cultural research on children’s television advertising in Hong Kong and Korea, the remaining Asian studies examined adult central figures in adult-focused television advertising. The values for each variable of gender representation for different Asian countries are presented in Table 3.

**Table 3:** Gender representation in Asian countries.

	Male – Female %														
Variables / Country (ies)	Bresnahan et al. (2001)			Furnham and Imadzu (2002)	Moon and Chan (2002)		Uray and Burnaz (2003)	Lee (2003)	Kim and Lowry (2005)	Das (2011)	Prieler and Centeno (2013)	Prieler et al. (2015)			Lim and Furnham (2016)
	Malaysia	Taiwan	Japan	Japan	Hong Kong	Korea	Turkey	Singapore	Korea	India	Philippines	Hong Kong	Japan	South Korea	Malaysia
Sex	57.14–42.86	46.01–53.99	43.35–56.65	56.34–43.66	54.40–45.60	48.40–51.60	46.50–53.50	23.91–76.09	42.18–57.82	56.80–43.20	41.70–58.30	59.50–40.50	59.20–40.80	61.10–38.90	28.68–71.32
Mode of presentation															
Disembodied Voice-over	81.0–19.0	81.0–19.0	68.0–32.0	67.0–34.19	–	–	–	71.90–28.10	20.5–9.80	80.40–10.0	–	–	–	–	–
Credibility basis															
Product user	–	–	–	19.50–46.45	10.20–4.10	6.10–7.10	53.40–74.40	54.50–45.70	–	60.10–70.30	–	–	–	–	61.50–82.10
Product authority	–	–	–	45.50–27.74	55.10–19.70	41.40–36.40	13.70–3.60	0.0–2.90	26.20–14.20	11.50–7.80	–	–	–	–	38.50–17.90
Role															
Dependent	–	–	–	19.50–56.77	57.50–65.70	58.70–62.90	44.30–64.70	0–25.70	21.30–37.50	32.0–52.40	–	–	–	–	56.80–85.0
Autonomous	–	–	–	–	42.50–34.30	41.30–37.10	–	–	–	68.0–47.60	–	–	–	–	–
Interviewer/Narrator	–	–	–	42.0–20.0	–	–	11.30–12.80	–	27.30–11.50	–	–	–	–	–	–
Location															
Domestic / indoor setting	15.0–26.0	21.0–21.0	23.0–41.0	21.50–33.55	–	–	30.80–57.70	0.0–28.60	37.20–21.30	21.60–37.30	47.10–61.40	38.30–51.20	30.10–44.90	37.40–70.90	27.80–46.40
Product argument															
Opinion-based	–	–	–	22.50–30.97	–	–	28.80–20.80	–	–	–	–	–	–	–	61.50–61.50
Fact-based	–	–	–	40.0–26.45	–	–	15.10–18.50	–	–	–	–	–	–	–	23.10–30.20
Product type															
Domestic products	54.0–54.0	51.0–72.0	47.0–74.0	59.0–69.03	–	–	55.50–66.70	36.40–40.0	–	64.30–79.30	71.70–71.0	76.40–86.80	53.30–67.60	40.40–80.30	94.80–88.70
Reward type															
Social enhancement	–	–	–	5.50–2.58	–	–	4.60–7.30	–	–	–	–	–	–	–	–
Self enhancement	–	–	–	23.50–30.97	–	–	16.10–44.40	–	–	–	–	–	–	–	32.40–49.0
Practical	–	–	–	6.50–7.10	–	–	16.10–18.50	–	–	–	–	–	–	–	2.90–9.40
Pleasure	–	–	–	28.50–27.74	–	–	26.40–9.70	–	–	–	–	–	–	–	35.30–17.70
End comment															
Present	–	–	–	55.0–40.0	–	–	93.80–96.40	–	–	–	–	–	–	–	–

The proportion of women in this study (48.90%) was higher than that found in advertisements from some Asian countries (Malaysia; Bresnahan et al., 2001; Japan; Furnham and Imadzu, 2002; Hong Kong and South Korea; Prieler et al., 2015), but lower than in other Asian countries (Taiwan; Bresnahan et al., 2001; Singapore; Lee, 2003; the Philippines; Prieler and Centeno, 2013; Turkey; Uray and Burnaz, 2003). The percentage of female voice-overs in this study (22.6%) was higher than that found in Malaysia, Taiwan (Bresnahan et al., 2001), and Korea (Kim and Lowry, 2005), while the percentage of male voice-overs (56.80%) was lower than the Asian countries’ average percentage (70%) for male characters (Furnham and Paltzer, 2010). However, this outcome highlights the stereotype in Asian cultures that female voice-overs, particularly those of young performers, are used for clarity, while male voices are used to demonstrate authority and expert knowledge (Furnham and Paltzer, 2010). The ratio of male-to-female product authority (74.80% of males and 35.30% of females) was higher in this study than in studies conducted in Japan, Turkey, Singapore, Malaysia, Hong Kong, and Korea (Furnham and Imadzu, 2002; Moon and Chan, 2002; Lee, 2003; Uray and Burnaz, 2003; Kim and Lowry, 2005; Lim and Furnham, 2016); whereas the ratio of male-to-female product users (16.50% of males and 63.90% of females) in this study was lower than that found in advertisements in Malaysia and Turkey (Uray and Burnaz, 2003; Lim and Furnham, 2016). However, this consistent pattern in television advertising of relegating women to the role of users (non-experts) while portraying men as authorities with product expertise promotes a negative stereotype among child viewers.

The proportion of men portrayed in narrator/interviewer roles (68%) in India was higher than that found in Turkey, Japan, and Korea (Furnham and Imadzu, 2002; Uray and Burnaz, 2003; Kim and Lowry, 2005). The frequency of women portraying dependent roles in this study (48.10%) was lower than that found in advertisements in several Asian countries (Japan; Furnham and Imadzu, 2002; Malaysia; Lim and Furnham, 2016; Hong Kong and Korea; Moon and Chan, 2002; Turkey; Uray and Burnaz, 2003). In the context of location, the proportion of women portrayed in domestic/home settings was relatively high (51.10%) compared to all Asian countries except Turkey, the Philippines, and South Korea (Uray and Burnaz, 2003; Prieler and Centeno, 2013; Prieler et al., 2015). Regarding product arguments, the findings for men favoring fact-based arguments (71.90%) and women favoring opinion-based arguments (55.60%) were higher than those for Japan (Furnham and Imadzu, 2002) and Turkey (Uray and Burnaz, 2003), but not those for Malaysia, where both men and women preferred opinion-based arguments (Lim and Furnham, 2016). This presumed inequality may indicate that women’s opinions are valued in traditional society because they rely on social networks and hierarchies for validation (Lim and Furnham, 2016), or that male voices are deemed more credible and persuasive, they are better suited to fact-based arguments (Gunter, 1990).

In addition, men and women were equally likely to advertise domestic products during children’s TV programming (69.80% vs. 87.20%), a finding contrary to that of other Asian studies (Furnham and Paltzer, 2010). This notable finding weakens the view that men are not associated with domestic items, and recent Asian studies indicate a shift in trends (Malaysia; Lim and Furnham, 2016; Philippines; Prieler and Centeno, 2013; Hong Kong; Prieler et al., 2015). The proportion of women appearing in domestic products (87.20%) was higher than that found in studies conducted in several Asian countries (Malaysia and Taiwan; Bresnahan et al., 2001; Japan; Furnham and Imadzu, 2002; Singapore; Lee, 2003; the Philippines; Prieler and Centeno, 2013; South Korea; Prieler et al., 2015; Turkey; Uray and Burnaz, 2003). As for rewards in this study, the findings suggested that for self-enhancement rewards, the proportion of men and women (37.40 vs. 68.40%) was higher than that in Japan, Malaysia, and Turkey (Furnham and Imadzu, 2002; Uray and Burnaz, 2003; Lim and Furnham, 2016). However, the use of practical rewards by men as a motivator (a weak indicator in sex stereotyping) was higher (23.0%) than that in studies conducted in Turkey, Japan, and Malaysia (Furnham and Imadzu, 2002; Uray and Burnaz, 2003; Lim and Furnham, 2016). Regarding end comments, the proportion of men and women (75.50% vs. 44.40%) was higher than that found in Japan (Furnham and Imadzu, 2002), but lower than that found in Turkey (Uray and Burnaz, 2003).

This study’s findings on children’s television advertising indicate a high level of gender stereotyping and corroborate previous researchers’ assessments of adult-focused television advertising in Asian countries (Neto and Pinto, 1998; Furnham and Mak, 1999; Furnham and Paltzer, 2010), demonstrating the concurrent validity of the McArthur and Resko coding scheme across multiple countries. For instance, in this study, men’s characters unconsciously reinforce dominance and authority over child viewers through their predominance in voice-overs, autonomous roles, end comments, and product authorities who deliver fact-based arguments. However, while women’s presence in diverse roles is positive, Indian advertising predominantly attempts to orient children to view female roles (i.e., conventional wives, mothers, and caregivers) in domestic settings. Therefore, female central figures are primarily depicted selling domestic, baby, and beauty products to female consumers, thus upholding traditional gender roles.

In a few instances, exciting differences are also noted between children’s television advertising and adult-focused advertisements from other Asian countries. For instance, in this study, Indian advertisers challenged women’s conventional role portrayals by increasing their representation in voice-overs, product authority, and autonomous roles. Simultaneously, men exhibit increased involvement with domestic products and rewards, such as self-enhancement, practical rewards, and pleasure rewards, thereby significantly increasing the role options available to children, regardless of gender. These variations in portrayal can be ascribed to the collectivist nature of Asian cultures, as television viewing in India is a family-oriented activity. In addition, 98% of Indian households own a single television, making co-viewing a significant factor for advertisers to effectively reach children and parents with non-children’s products [e.g., consumer durables, e-commerce, banking, and insurance; KPMG (Klynveld Peat Marwick Goerdeler), 2018]. This noteworthy phenomenon could potentially account for the increased representation of men and women in non-stereotypical roles in children’s television advertisements.

### 4.2. Theoretical and practical implications

According to role congruity theory (Eagly and Karau, 2002), perpetuating distorted stereotypes in the media without a full palette of roles for males and females may hinder children’s ability to learn about their gender identities because they may see limited diversification in their social roles. Similarly, the consistent pattern of associating women with self-enhancement rewards may cast a lopsided gender representation on observers, particularly girls, as they may begin to accept these stereotypes as social norms and act accordingly (Ganahl et al., 2003). Moreover, according to social learning theory, when there is an enticing model whose behavior is rewarded, children seek to attain these patterns of behavior more ardently, which may reinforce viewers’ gender-stereotyped occupational schemas (Bandura, 1969; Smith, 1994). The supposed gender imbalance in television advertising is problematic, but this study’s findings provide essential information to marketers and parents regarding the prevalent advertising practices in India. Gender is the primary segmentation variable from the advertiser’s perspective when developing marketing strategies, designating target groups, and portraying a specific gender as a user of particular products (An and Kim, 2007). However, public policy regulators, activists, and advertising councils contend that advertisers create and perpetuate gender stereotypes, which can undermine gender equality and harm society (Oppliger, 2007). Children are a protected segment of consumers, and the visual images they observe during their formative years shape their identities. Consequently, it should be the shared responsibility of parents, advertisers, advertising researchers, and public policymakers to ensure their protection from deceptive images and stereotypes.

Several nations have begun to invest in advertising education programs to increase children’s critical attitudes and resistance to the persuasive power of advertising (Rozendaal et al., 2016). From this perspective, it is imperative to encourage gender-equitable practices by complementing product advertising with gender-equal and gender-neutral portrayals without voicing gender preferences. In addition, marketers and advertisers should evaluate the totality of the message, emphasizing the choice of a representative gender for advertisements directed at children. For example, facilitating active parenthood responsibilities by emphasizing men’s participation in household activities, such as cooking, cleaning, dietary choices, and nurturing actions, would be a progressive step toward demonstrating that fathers are also involved in decisions concerning children and the household. Similarly, the increased representation of women in different roles within and outside the home, involving leadership, independence, and career-oriented roles, could expand children’s aspirations to explore positive gender options. Thus, in a country where 98% of Indian households have a minimum of one television, portraying adults in more egalitarian gender roles in children’s television advertising becomes even more crucial for advertisers considering future marketing opportunities via this medium.

## 5. Conclusion

This study examined traditional stereotypical portrayals of men and women in the context of Indian children’s television advertising for almost all investigated variables. However, the results revealed fewer stereotypical elements, such as females progressively increasing their presence in a few male-dominated categories, whereas males were portrayed positively in egalitarian roles in some other categories. It can be surmised that current portrayals mirror social norms and the target market’s interests; however, they echo advertisers’ recognition of the importance of counter-stereotyping in recent years, thereby delimiting gender-based differences.

However, this study has some limitations. Similar previous studies used cross-sectional samples of TV advertisements from top-grossing cartoon channels; however, including regional cartoon channels with varying timeslots in a longitudinal analysis might boost the sample’s representativeness. Concurrent replication of the same study on adult-focused and children’s advertising would assist researchers in identifying the various creative advertising strategies used by advertisers to target distinct audience segments within the same country. For the purpose of comparing the results with those of other studies, only adults were considered for coding purposes, and children were excluded as central characters, which is a limitation that can be addressed by including them in future research. Their inclusion may provide substantially different results for gender representation in television advertising in a particular country. While drawing broad generalizations from a single study is challenging, the findings add value to international marketers by offering insights into the overall level of gender portrayals in South Asian countries such as India. The findings strongly suggest that Indian children consume highly gender-stereotypical content in television advertising on cartoon channels, and when such standardization extends to other media, its repercussions can be detrimental and irreversible if advertisers and policy makers do not intervene. Consequently, there is sufficient evidence to validate the concerns of many parents, and future research should focus on the probable effects of gender representation on child audiences.

## Data availability statement

The raw data supporting the conclusions of this article will be made available by the authors, without undue reservation.

## Author contributions

CKR: conceptualization, methodology, validation, formal analysis, investigation, and writing – original draft. SN: supervision, validation, and writing – reviewing and editing. All authors contributed to the article and approved the submitted version.

## Conflict of interest

The authors declare that the research was conducted in the absence of any commercial or financial relationships that could be construed as a potential conflict of interest.

## Publisher’s note

All claims expressed in this article are solely those of the authors and do not necessarily represent those of their affiliated organizations, or those of the publisher, the editors and the reviewers. Any product that may be evaluated in this article, or claim that may be made by its manufacturer, is not guaranteed or endorsed by the publisher.

## Abbreviations

BARC: Broadcast Audience Research Council
IBEF: India Brand Equity Foundation
KPMG: Klynveld Peat Marwick Goerdeler
MoSPI: Ministry of Statistics and Programme Implementation Government of India
